# Supplementary material for: Cytosolic Isocitrate Dehydrogenase from Arabidopsis thaliana Is Regulated by Glutathionylation
Source: Antioxidants (Basel). 2019 Jan 8;8(1):16. doi: 10.3390/antiox8010016 (PMC6356969; doi:10.3390/antiox8010016)
Supplement: Supplementary file 1 [file antioxidants-08-00016-s001.zip › Suppl Figure S1.pptx]

## Slide 1
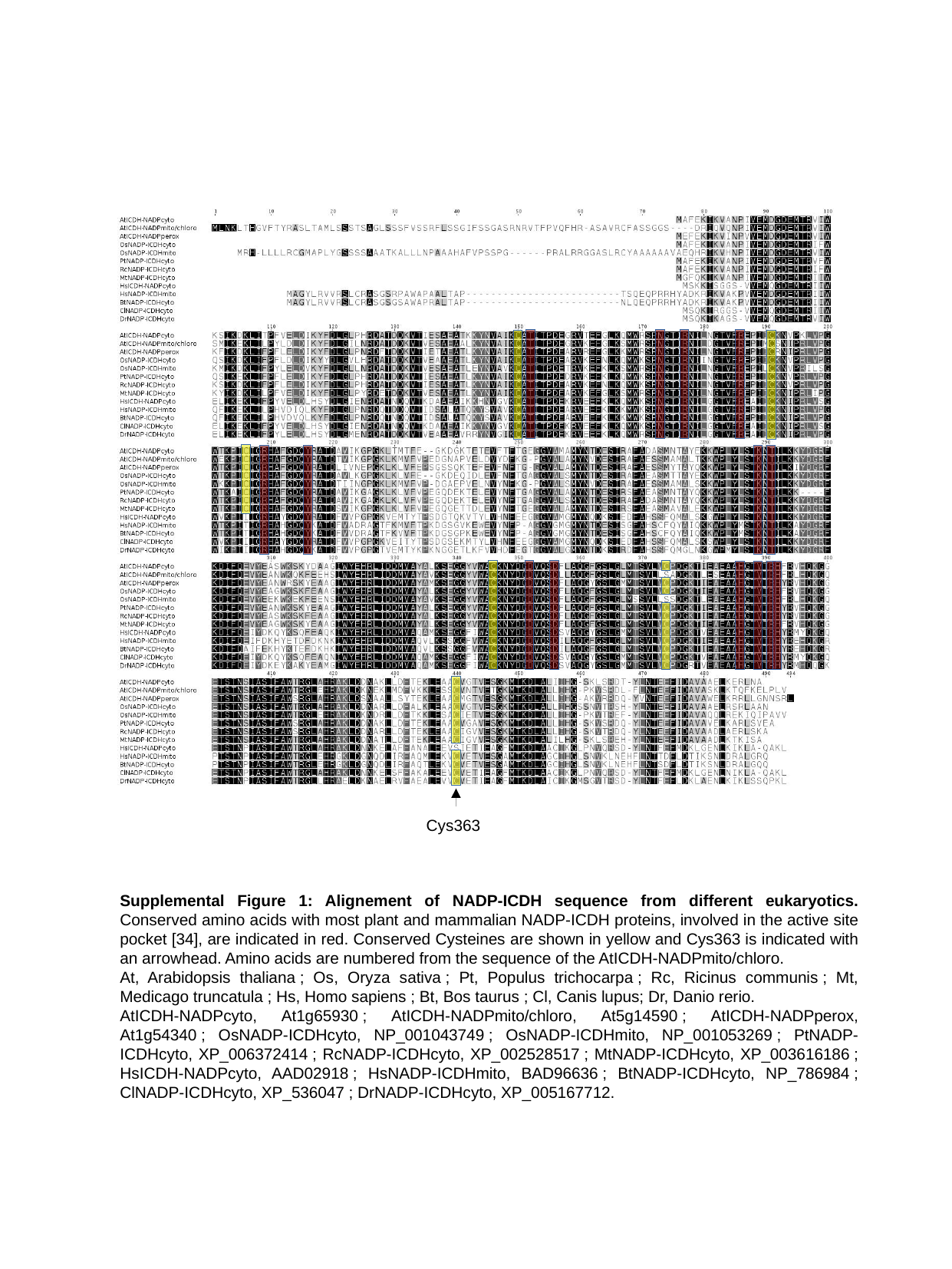

Cys363
Supplemental Figure 1: Alignement of NADP-ICDH sequence from different eukaryotics. Conserved amino acids with most plant and mammalian NADP-ICDH proteins, involved in the active site pocket [34], are indicated in red. Conserved Cysteines are shown in yellow and Cys363 is indicated with an arrowhead. Amino acids are numbered from the sequence of the AtICDH-NADPmito/chloro.
At, Arabidopsis thaliana ; Os, Oryza sativa ; Pt, Populus trichocarpa ; Rc, Ricinus communis ; Mt, Medicago truncatula ; Hs, Homo sapiens ; Bt, Bos taurus ; Cl, Canis lupus; Dr, Danio rerio.
AtICDH-NADPcyto, At1g65930 ; AtICDH-NADPmito/chloro, At5g14590 ; AtICDH-NADPperox, At1g54340 ; OsNADP-ICDHcyto, NP_001043749 ; OsNADP-ICDHmito, NP_001053269 ; PtNADP-ICDHcyto, XP_006372414 ; RcNADP-ICDHcyto, XP_002528517 ; MtNADP-ICDHcyto, XP_003616186 ; HsICDH-NADPcyto, AAD02918 ; HsNADP-ICDHmito, BAD96636 ; BtNADP-ICDHcyto, NP_786984 ; ClNADP-ICDHcyto, XP_536047 ; DrNADP-ICDHcyto, XP_005167712.
