# Supplementary material for: Cytosolic Isocitrate Dehydrogenase from Arabidopsis thaliana Is Regulated by Glutathionylation
Source: Antioxidants (Basel). 2019 Jan 8;8(1):16. doi: 10.3390/antiox8010016 (PMC6356969; doi:10.3390/antiox8010016)
Supplement: Supplementary file 1 [file antioxidants-08-00016-s001.zip › Suppl Figure S2.pptx]

## Slide 1
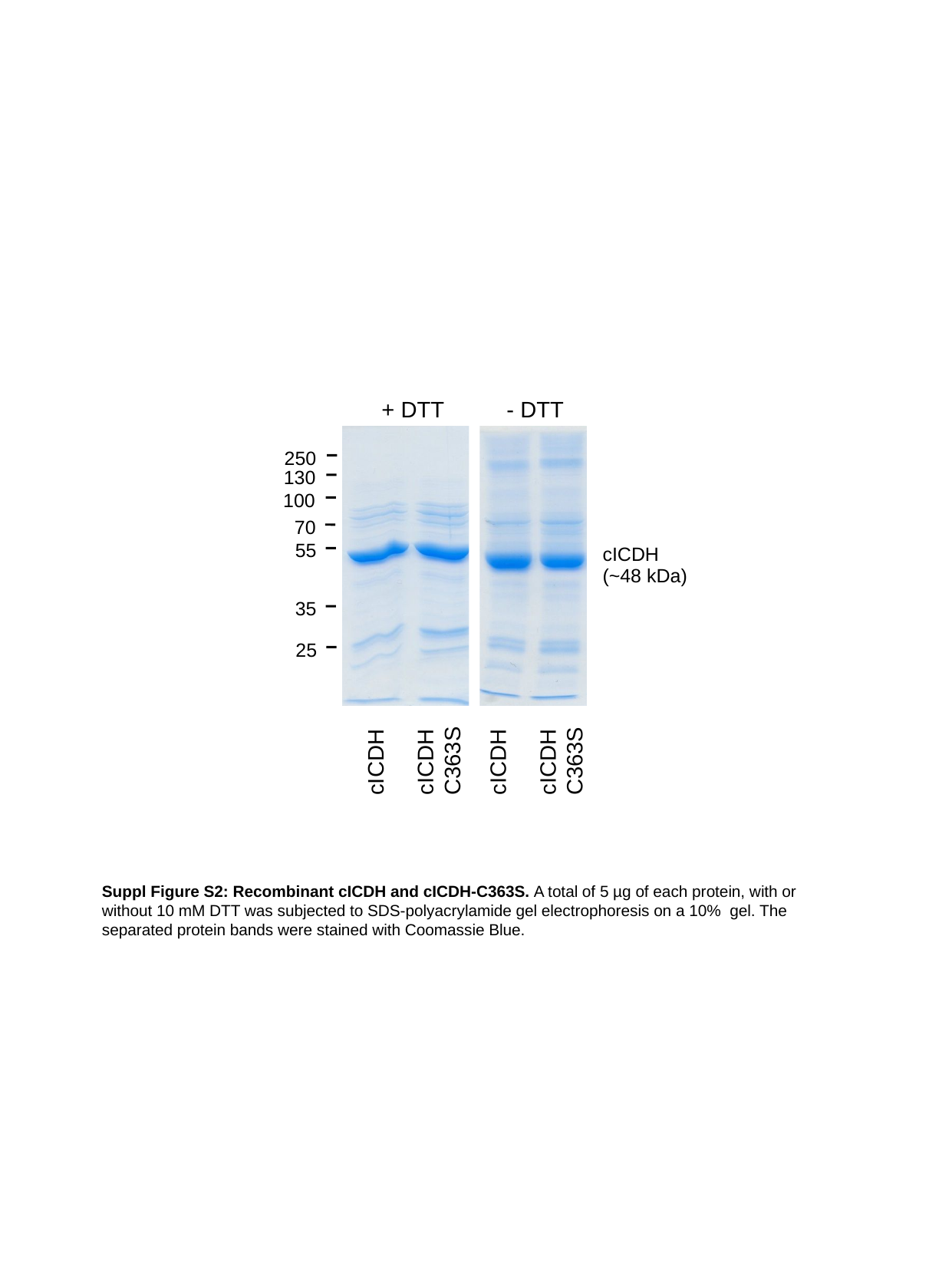

+ DTT
 - DTT
250
130
100
70
55
35
25
cICDH
(~48 kDa)
cICDH
C363S
cICDH
C363S
cICDH
cICDH
Suppl Figure S2: Recombinant cICDH and cICDH-C363S. A total of 5 µg of each protein, with or without 10 mM DTT was subjected to SDS-polyacrylamide gel electrophoresis on a 10% gel. The separated protein bands were stained with Coomassie Blue.
