# Supplementary material for: Cytosolic Isocitrate Dehydrogenase from Arabidopsis thaliana Is Regulated by Glutathionylation
Source: Antioxidants (Basel). 2019 Jan 8;8(1):16. doi: 10.3390/antiox8010016 (PMC6356969; doi:10.3390/antiox8010016)
Supplement: Supplementary file 1 [file antioxidants-08-00016-s001.zip › Suppl Figure S3.pptx]

## Slide 1
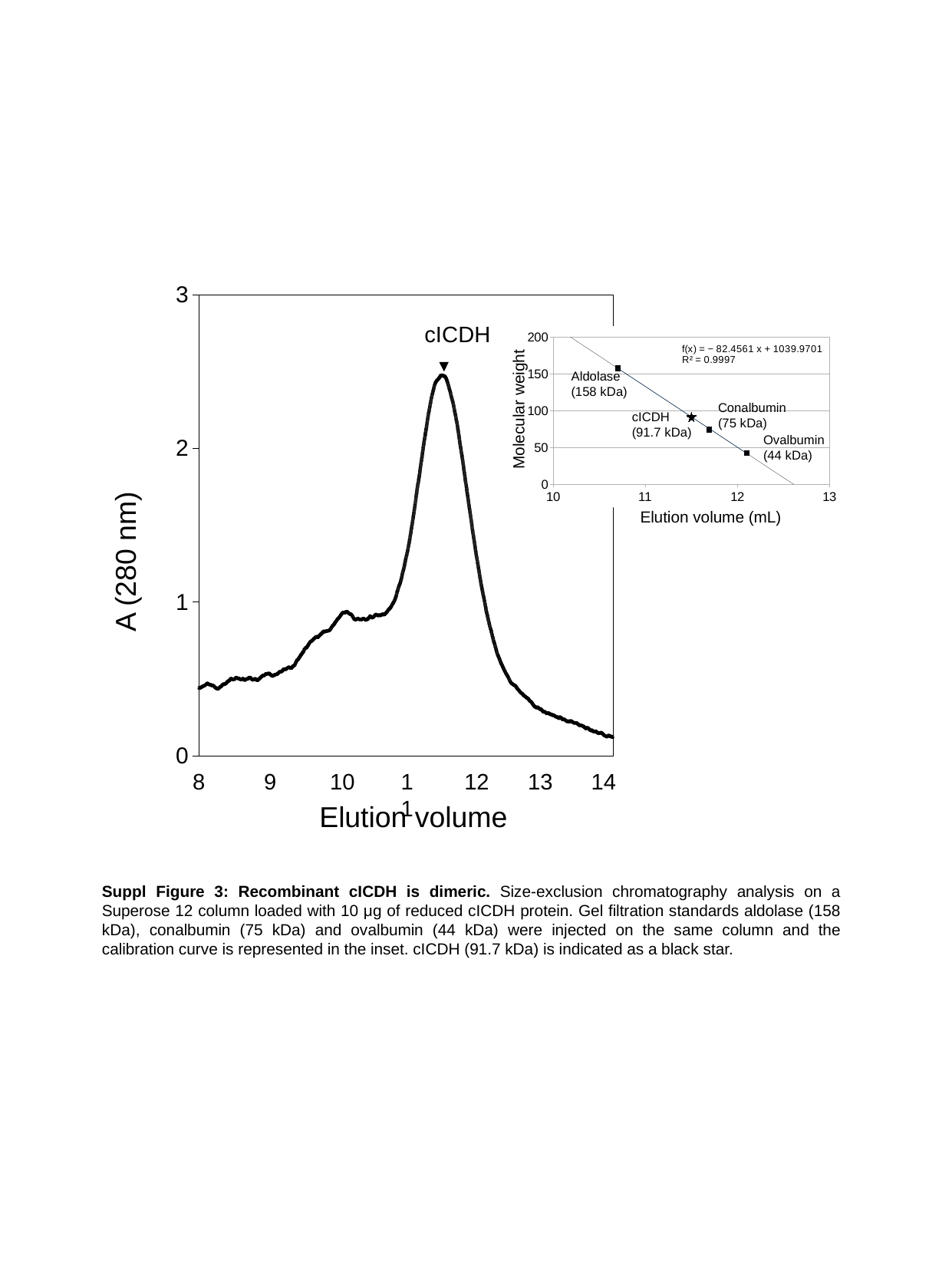

cICDH
Aldolase
(158 kDa)
Conalbumin
(75 kDa)
Molecular weight
cICDH
(91.7 kDa)
Ovalbumin
(44 kDa)
Elution volume (mL)
A (280 nm)
8
9
10
11
12
13
14
Elution volume
Suppl Figure 3: Recombinant cICDH is dimeric. Size-exclusion chromatography analysis on a Superose 12 column loaded with 10 μg of reduced cICDH protein. Gel filtration standards aldolase (158 kDa), conalbumin (75 kDa) and ovalbumin (44 kDa) were injected on the same column and the calibration curve is represented in the inset. cICDH (91.7 kDa) is indicated as a black star.
