# Supplementary material for: Cytosolic Isocitrate Dehydrogenase from Arabidopsis thaliana Is Regulated by Glutathionylation
Source: Antioxidants (Basel). 2019 Jan 8;8(1):16. doi: 10.3390/antiox8010016 (PMC6356969; doi:10.3390/antiox8010016)
Supplement: Supplementary file 1 [file antioxidants-08-00016-s001.zip › Suppl Figure S4.pptx]

## Slide 1
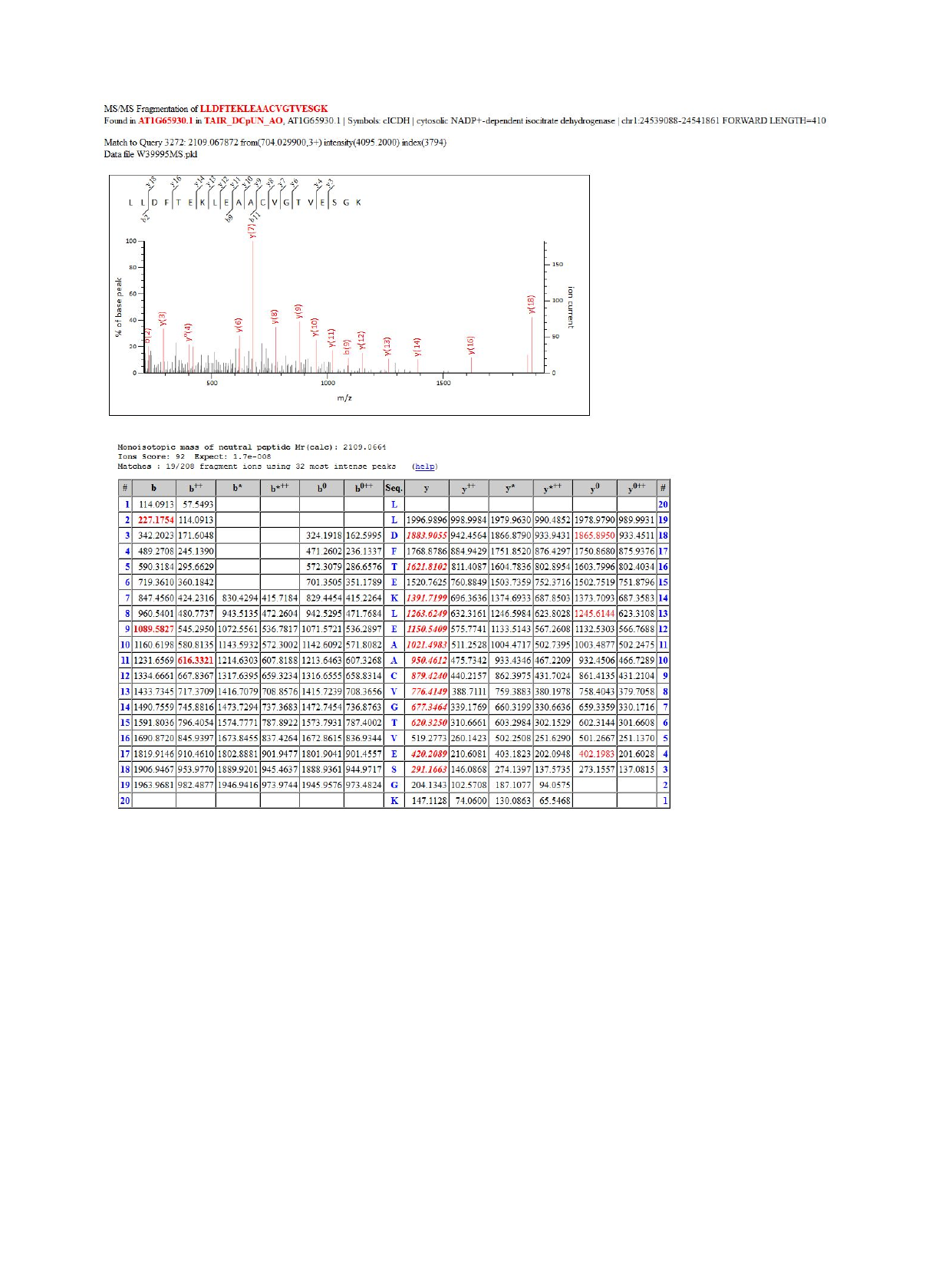

## Slide 2
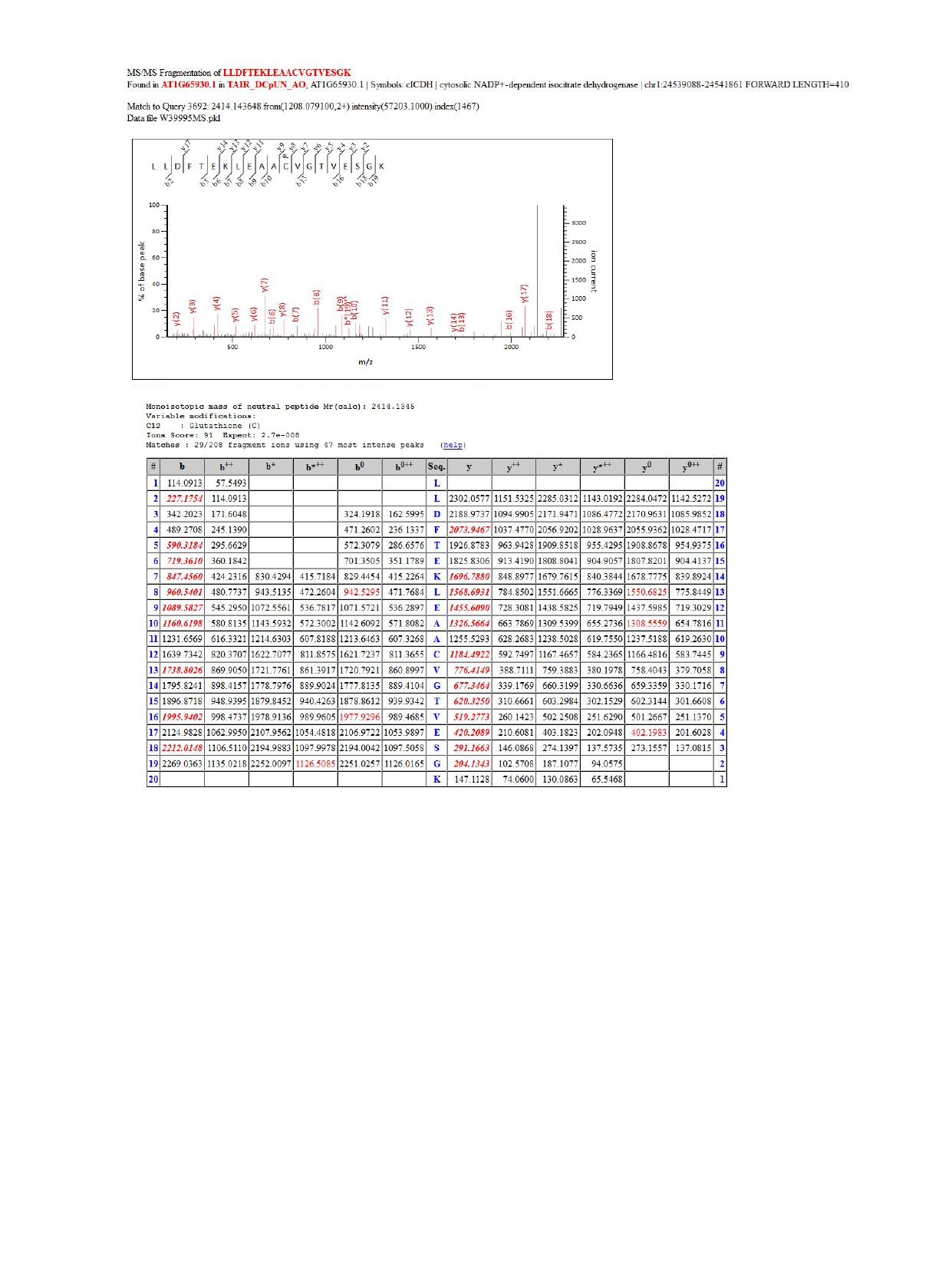

## Slide 3
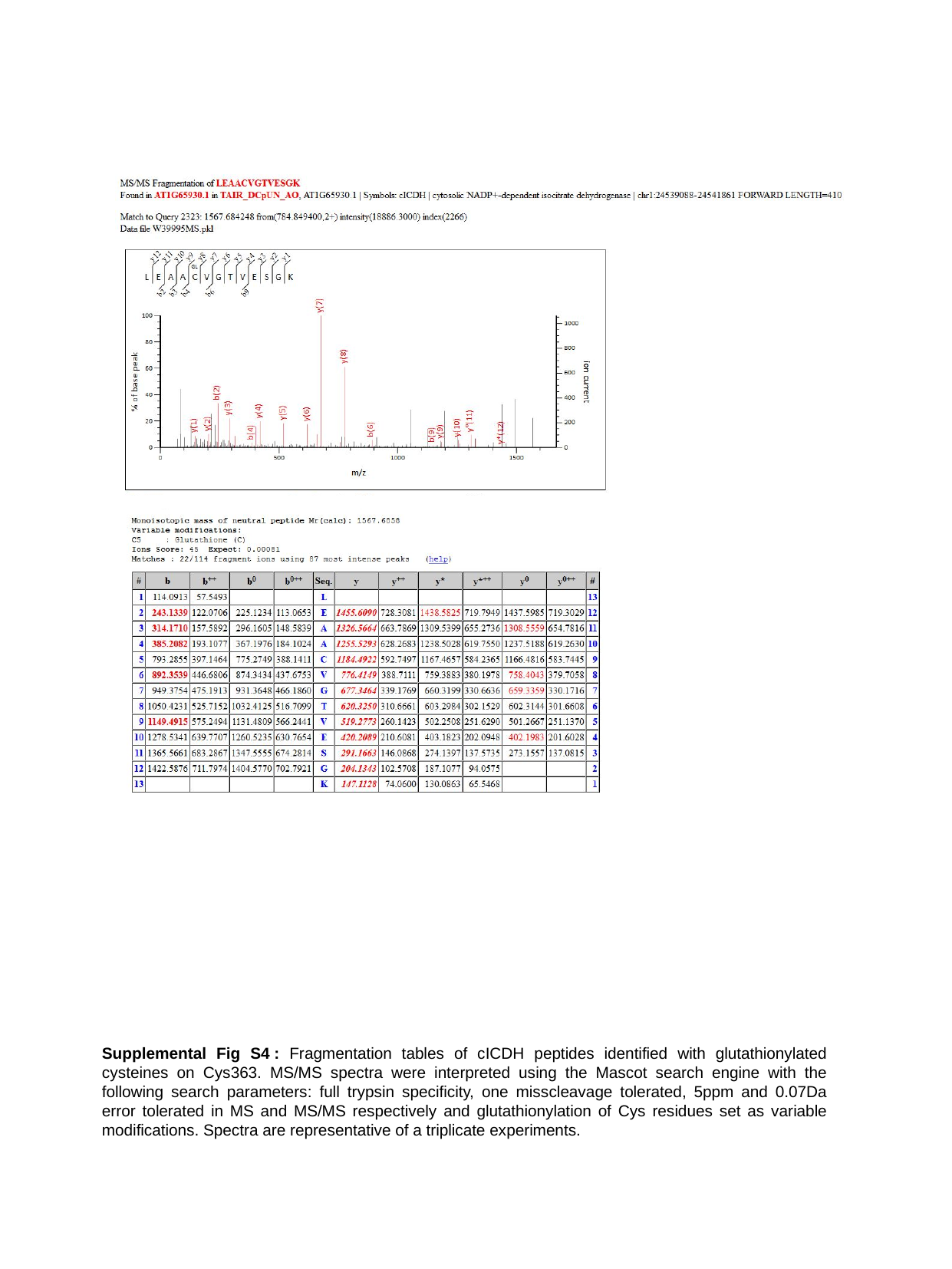

Supplemental Fig S4 : Fragmentation tables of cICDH peptides identified with glutathionylated cysteines on Cys363. MS/MS spectra were interpreted using the Mascot search engine with the following search parameters: full trypsin specificity, one misscleavage tolerated, 5ppm and 0.07Da error tolerated in MS and MS/MS respectively and glutathionylation of Cys residues set as variable modifications. Spectra are representative of a triplicate experiments.
