# Supplementary material for: Cytosolic Isocitrate Dehydrogenase from Arabidopsis thaliana Is Regulated by Glutathionylation
Source: Antioxidants (Basel). 2019 Jan 8;8(1):16. doi: 10.3390/antiox8010016 (PMC6356969; doi:10.3390/antiox8010016)
Supplement: Supplementary file 1 [file antioxidants-08-00016-s001.zip › Suppl Figure S5.pptx]

## Slide 1
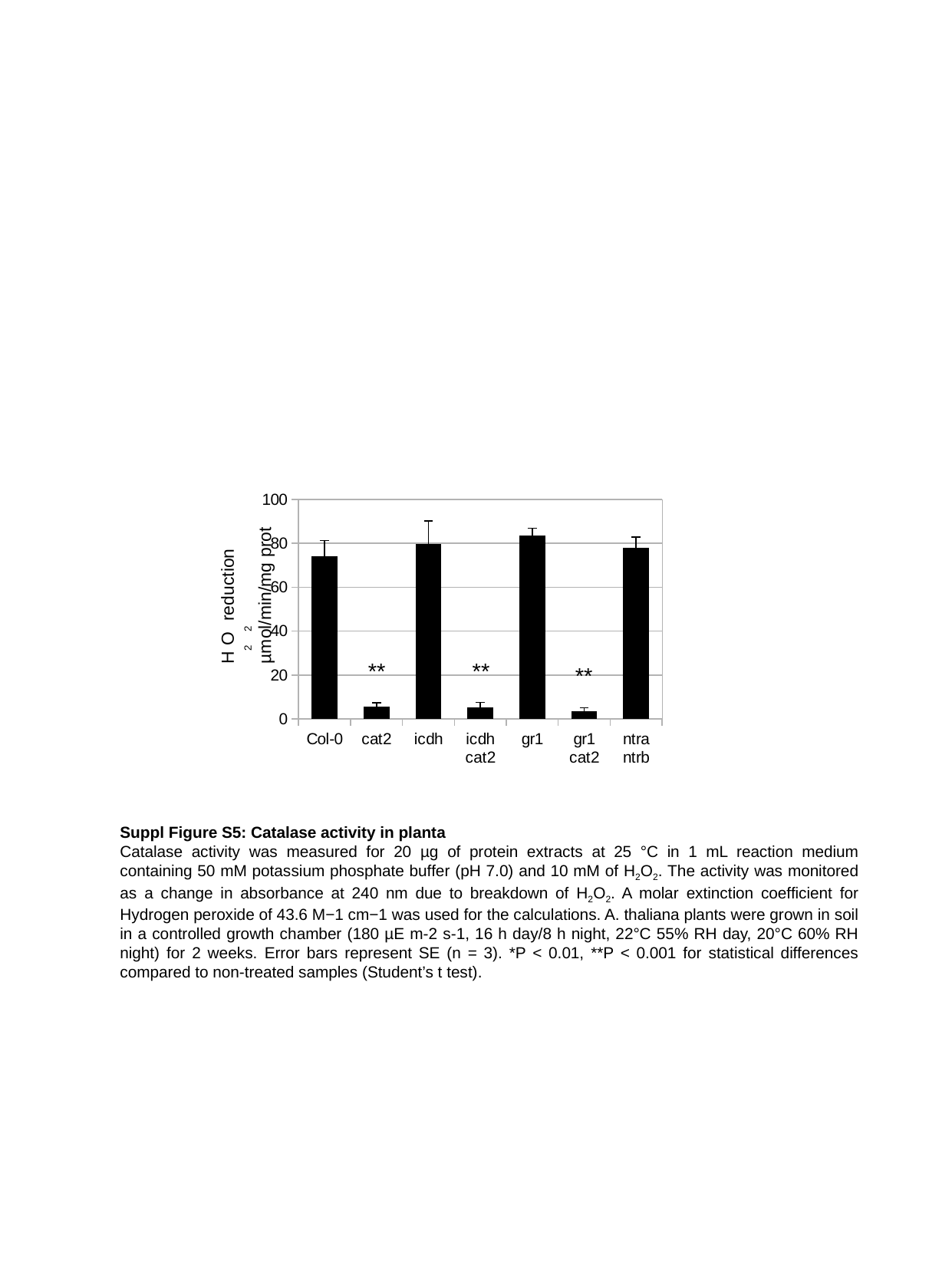

### Chart
| Category | Column Y |
|---|---|
| Col-0 | 73.97 |
| cat2 | 5.73 |
| icdh | 79.7 |
| icdh cat2 | 5.16 |
| gr1 | 83.72 |
| gr1 cat2 | 3.44 |
| ntra ntrb | 77.98 |H2O2 reduction
µmol/min/mg prot
**
**
**
Suppl Figure S5: Catalase activity in planta
Catalase activity was measured for 20 µg of protein extracts at 25 °C in 1 mL reaction medium containing 50 mM potassium phosphate buffer (pH 7.0) and 10 mM of H2O2. The activity was monitored as a change in absorbance at 240 nm due to breakdown of H2O2. A molar extinction coefficient for Hydrogen peroxide of 43.6 M−1 cm−1 was used for the calculations. A. thaliana plants were grown in soil in a controlled growth chamber (180 µE m-2 s-1, 16 h day/8 h night, 22°C 55% RH day, 20°C 60% RH night) for 2 weeks. Error bars represent SE (n = 3). *P < 0.01, **P < 0.001 for statistical differences compared to non-treated samples (Student’s t test).
