# Supplementary material for: Cytosolic Isocitrate Dehydrogenase from Arabidopsis thaliana Is Regulated by Glutathionylation
Source: Antioxidants (Basel). 2019 Jan 8;8(1):16. doi: 10.3390/antiox8010016 (PMC6356969; doi:10.3390/antiox8010016)
Supplement: Supplementary file 1 [file antioxidants-08-00016-s001.zip › Suppl Figure S6.pptx]

## Slide 1
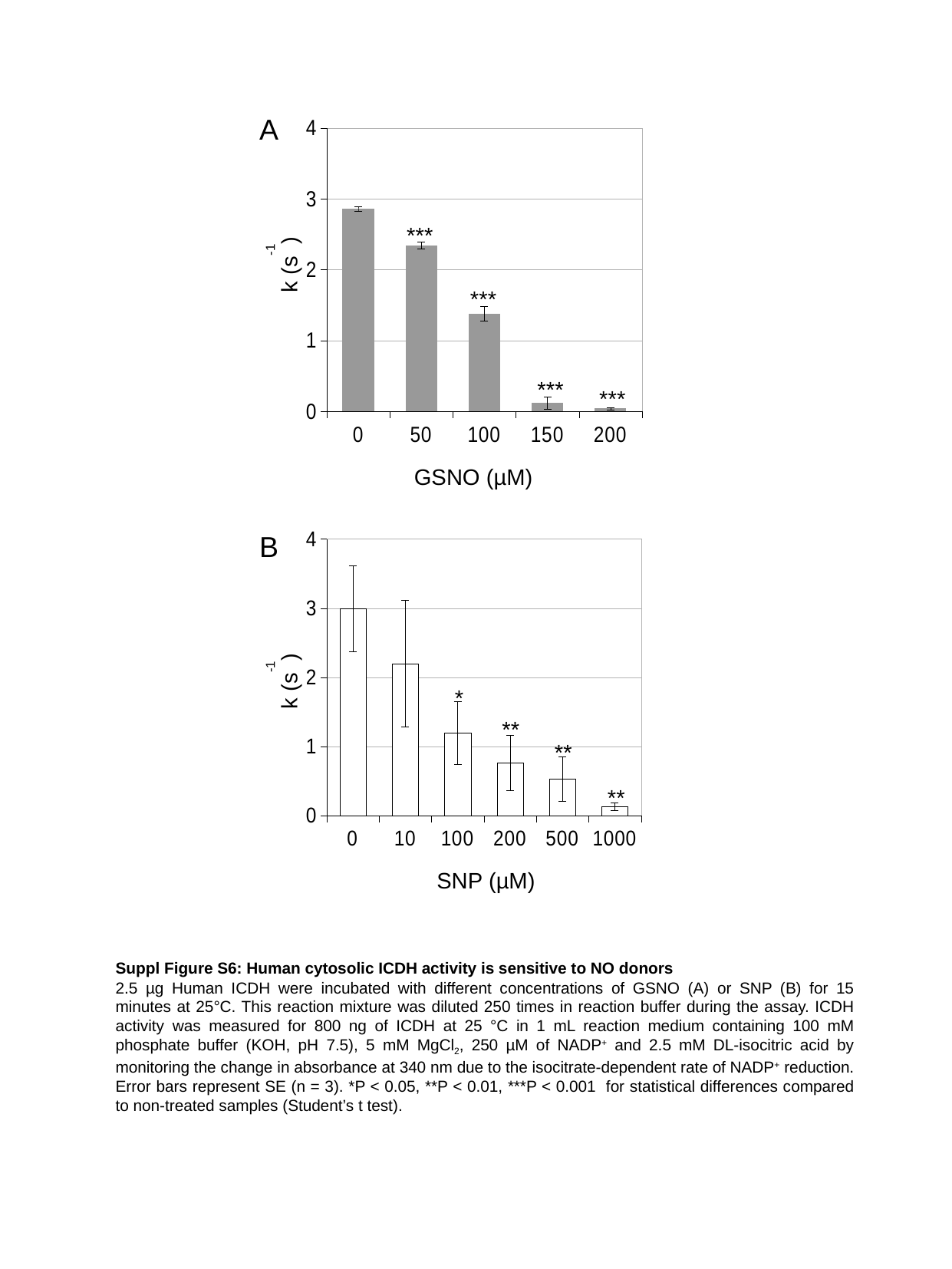

A
***
k (s-1)
***
***
***
GSNO (µM)
B
k (s-1)
*
**
**
**
SNP (µM)
Suppl Figure S6: Human cytosolic ICDH activity is sensitive to NO donors
2.5 µg Human ICDH were incubated with different concentrations of GSNO (A) or SNP (B) for 15 minutes at 25°C. This reaction mixture was diluted 250 times in reaction buffer during the assay. ICDH activity was measured for 800 ng of ICDH at 25 °C in 1 mL reaction medium containing 100 mM phosphate buffer (KOH, pH 7.5), 5 mM MgCl2, 250 µM of NADP+ and 2.5 mM DL-isocitric acid by monitoring the change in absorbance at 340 nm due to the isocitrate-dependent rate of NADP+ reduction.
Error bars represent SE (n = 3). *P < 0.05, **P < 0.01, ***P < 0.001 for statistical differences compared to non-treated samples (Student’s t test).
