# Supplementary material for: Cytosolic Isocitrate Dehydrogenase from Arabidopsis thaliana Is Regulated by Glutathionylation
Source: Antioxidants (Basel). 2019 Jan 8;8(1):16. doi: 10.3390/antiox8010016 (PMC6356969; doi:10.3390/antiox8010016)
Supplement: Supplementary file 1 [file antioxidants-08-00016-s001.zip › suppl Table S1.pdf]

Supplemental Table S1: Primers used in this study.

Primer Name	Primer Sequence	Experiment Purpose
cICDH-NdeI	GCCATATGGCGTTTGAGAAGATCAAG	Cloning cICDH in pET-16b
cICDH-BamH1	CGGGATCCTTAAGCATTGAGTCTTTCTTTGAG	Cloning cICDH in pET-16b
cICDH-C363S-F	AAGCTGGAAGCCGCTAGTGTTGGGACAGTGG	Point mutation of cICDH-C363S
cICDH-C363S-R	CCACTGTCCCAACACTAGCGGCTTCCAGCTT	Point mutation of cICDH-C363S
